# NGOs’ experiences of navigating the open access landscape

**DOI:** 10.12688/f1000research.17359.2

**Published:** 2019-10-18

**Authors:** Nilam McGrath

**Affiliations:** 1 www.nilammcgrath.com @TalkingEvidence, Leeds, UK

**Keywords:** Open Access, NGOs, Global South, global development, funders, publishers, low middle income country, Nepal, Bangladesh, China, Pakistan, Swaziland, Ghana

## Abstract

Grant-led consortia working in the global development sector rely on the input of local and national non-government organisations in low- and middle-income countries. However, the open access mandates and mechanisms embedded within grants and promoted by funders and publishers are designed almost exclusively with large universities and research institutions in mind. Experiences from the consortium of health research non-government organisations comprising the Communicable Diseases Health Service Delivery research programme show that implementing open access mandates is not as simple or frictionless as it initially appears.

## Introduction

Grants aimed at tackling issues prevalent within low- and middle-income countries (LMICs) often encourage collaboration or consortium working between Northern universities and organisations in the Global South, thereby bringing together research institutions, non-government organisations (NGOs), the private sector and government agencies.

National NGOs in the Global South are particularly important to the research process as their responsiveness to national agendas makes them ideal global development partners, making them crucial drivers of research design and implementation. In many research programmes, it is their labour, their insight, their networks and their relationships that influence how an intervention will be received and taken up in their country context. This makes NGOs extremely powerful actors in the research evidence production cycle.

Large, collaborative grants now come with open access (OA) mandates to aid research uptake. Many funders from the Global North (for example Wellcome Trust, UK Research and Innovation, and the Department for International Development) are clear about the mechanisms by which Northern university-led research programmes can make their work available via OA mechanisms. Similarly, scholarly publishers have mechanisms in place to grant waivers for article processing charges (APCs) to aid funders’ OA mandates.

However, existing OA mechanisms are overwhelmingly geared towards supporting universities and large research institutions in the Global North to share research outputs, with the assumption that all collaborating partners, including NGO partners based in LMICs, are able to adequately adapt their working practices to meet OA requirements. In an effort to meet OA requirements, this results in NGO partners funnelling their efforts through Northern universities, thereby creating an over-reliance on their Northern partners to, firstly, access evidence to inform and shape their country-specific research interventions, and secondly, to publish new research they have generated within their country setting. This over-reliance inevitably results in stalled or slower research design processes, information bottlenecks, increased bureaucracy when attempting to publish papers and unanticipated costs.

## An over-reliance on Northern universities to access journal articles

Working at a Northern university undoubtedly has its research access privileges; it gives employees (academic researchers, undergraduates, postgraduates, operational and administration staff) unparalleled access to journal articles through its library. NGO partners and their researchers based in LMICs know this, and many rely on their university counterparts to help them access relevant literature and research findings so that they can then design their country-specific research interventions. This creates a dependency on Northern universities by Southern NGO partners to access information. Furthermore, it generates frequent and one could argue, unnecessary requests for information to Northern universities, creating information bottlenecks and stalling the research process at stages where evidence is needed.

It also results in Northern universities and their researchers inadvertently becoming gatekeepers of information. Take an example from my own work which involved writing up the results of a media monitoring exercise in Nepal. My colleague in Nepal was relying on my privileged access at a UK university to search for similar and contrasting studies, which I would then send to him in Nepal to review and synthesise. Aside from being time-consuming, this raises questions about my suitability and ability as a non-Nepali, Northern-based researcher to accurately spot and explore the nuances and contextual issues of a particular topic that only my colleague would recognise when searching academic and grey literature.

This type of back-and-forth between Southern and Northern researchers occurs more than we realise in operational research and in consortia that rely on NGO partnerships. The information dependency that it promotes decreases the exposure that NGOs in LMICs have to larger bodies of research and it has huge implications for building the capacity of individuals and organisations working in LMICs to independently access, interpret, synthesise, generate and share their research evidence. The nature of this dependency is at the core of debates surrounding decolonising development and structural inequalities in knowledge production and sharing ( Bonaccorso *et al.*, 2014; Chan *et al*., 2011) (see the work of the Open and Collaborative Science in Development Network and The Knowledge Gap). Failure to address access privileges and their associated power further embeds structural inequality within South-North partnerships.

## But they’ve got Research4Life, right?

Lack of access can be countered somewhat with initiatives such as Research4Life, but this is not the panacea that many claim ( Chan *et al*., 2011; Smith *et al.*, 2017; Zachariah *et al*., 2014). Research4Life offers 3 types of access: 1) free access to papers restricted to NGOs in Group A countries only; 2) low-cost access for NGOs based in Group B countries, costing between US$1000 - US$1500 annually to access the same content; and 3) a ‘transitional path offer’ for countries no longer eligible for free or low-cost access, costing US$1500 annually for access to select journals depending on organisation type.

There is, however, a fourth type of access for NGOs: no access. Any blanket exclusion of NGOs from countries deemed to be based in more affluent countries, for example in China, assumes that NGOs are in the same stable economic position as their established, affluent national counterparts. The assumptions embedded in the low-cost, transition and no access scenarios do not acknowledge that the non-profit sector works to tight programme budgets and restricted funding. Indeed, restricted or conditional funding is the norm for the global development sector within which NGOs operate. As Smith *et al.* (2017) state, “…certain institutions – notably those that play many other roles such as healthcare provision and health prevention and promotion – have competing claims for limited funding” (2017:2). Access to new and existing research via paid subscriptions or annual fees, therefore, remains low on the list of priorities for NGOs, who instead are committed to using grant funding to pay solely for field research, staff time and other implementation costs ( Smith *et al*, 2017). For ‘transitional’ countries, for example, the Philippines or Indonesia, the same assumption applies; NGOs in these countries are financially sustainable and therefore able to pay US$1500 annually for accessing research.

So, with limited budgets, and with Research4Life out of reach, where do NGOs go when they need to access research legally? That’s right – their colleagues at Northern universities. And so, the dependency cycle repeats itself, with this South to North pipeline of access becoming the default option for many grant collaborations and consortia.

The illegal route involves accessing journals via sites such as Sci-Hub, and judging by Sci-Hub’s download statistics, it is a popular alternative ( Bohannon, 2016) particularly for LMICs; China, India and Iran were the top 3 of downloading countries in 2016 ( Hull, 2016). It’s also an alternative that places researchers in an ethical dilemma ( Bendezú-Quispe *et al.,* 2016).

## Northern universities’ role in publishing papers

In consortia working, the process of publishing papers is also often funnelled through a Northern university partner. On paper, this would appear to be a logical and straight forward process, but experiences from the Communicable Diseases Health Service Delivery (COMDIS-HSD) research programme show that this is not the frictionless process that publishers like to promote. There is almost always an issue with securing waivers, with NGOs often resorting to time-consuming and bureaucratic workarounds to secure waivers for APCs. Prior research has also shown a lack of transparency in the number of waivers being granted ( Burchardt, 2014; Peterson *et al.*, 2013)

When publishing journal articles, APCs are the most obvious cost for all grant recipients. Waivers are designed to counter these costs, but the COMDIS-HSD experience was that the process for securing an APC waiver was inconsistent across all publishers ( Lawson, 2015; Smith *et al.*, 2017). Inconsistencies stem from whether it is the country of the ‘corresponding author’ or the ‘first author’ that is being used as a proxy to determine whether a waiver is granted. Typically, it is either one or the other – corresponding or first author – but some publishers are prepared to grant a waiver if just one co-author is from a LMIC. PLOS determine waivers according to where the funding institution is based. The inconsistency across publishers can lead to last minute changes in the authorship list in order to meet publishers’ criteria and secure a waiver, i.e. a swapping of corresponding and first author names.

At COMDIS-HSD, all papers were co-authored by both UK and LMIC-based authors. Corresponding authors were usually UK-based, as this helped expedite APCs using a UK university corporate credit card, thereby alleviating the financial burden of high APC payments for their NGO partners. However, publishers incorrectly viewed the UK base of a corresponding author as an indication of their ability to pay the full open access APC. The consortium then had to enter into lengthy correspondences with several journals to, firstly, prove that the majority of their authors were from LMICs (and therefore automatically entitled to a waiver), and secondly, to secure the waiver in writing. This back and forth to secure waivers is not uncommon for authors based in LMICs. In short, authors continually have to prove how ‘poor’ they are. Furthermore, using UK-based corresponding authors for COMDIS-HSD wasn’t without consequence, as these authors were charged an additional value added tax of 20% of the original APC; an unavoidable addition as this is a tax levied by the UK government.

## The unanticipated costs of publishing journal articles

Additionally, COMDIS-HSD encountered unanticipated costs and hidden charges whilst navigating the open access process, for example, USD$50/£50, for using invoicing rather than direct online payment systems. The issue here is not the cost of APCs or invoicing per se, but the lack of transparency on publishers’ websites that these costs exist. Authors often have to navigate away from the journals’ homepage to find APCs, waiver processing information and other caveats, once again adding unnecessary time and bureaucracy to what should be relatively swift submissions, waiver applications, and payment processes.

There is a fine balance between ensuring research outputs are made available quickly and giving people adequate time to pay APCs. The nature of NGO work means that authors are often conducting field research, meaning that email access is limited and that demands for APC payments can go unnoticed for days or weeks, missing the 7 or 14-day payment deadline of some publishers. Once received, the payment request may be forwarded to a paying institution (the University of Leeds in the case of COMDIS-HSD), adding more time to the payment process. In such cases where short payment deadlines are missed, publishers automatically add additional charges for not being able to pay APCs within 7–14 days; an unreasonably short time, given that most companies allow 30 days for payment.

In one instance in July 2018, Springer Nature instructed COMDIS-HSD to pay an APC online within 7 days. If the APC was not paid after 7 days, an invoice would be issued and an administration charge of £50/EUR55/USD$75 added to the APC, with no option to revert to online payment thereafter. This was in spite of a 30-day payment deadline being advertised on their website. My requests for extending the deadline while we waited for the relevant conference discount code (from administrators at BMC Health Services Research, owned by Springer Nature), and so that the university’s finance department could authorise the payment (which can take 1–3 working days) were met with further reminders to pay within 7 days. Pleading phone calls and emails to university finance staff and conference organisers meant that we were able to pay the invoice on Day 7, but why would any publisher subject any author to this in the first place?

To avoid additional ‘late payment’ fees and to meet short payment deadlines such as this, authors will often attempt to pay on personal credit cards; an unreasonable situation for any individual to be placed in. What this ultimately amounts to is NGOs in LMICs being forced to adhere to payment schedules and processes that make them more financially vulnerable than their larger, established, university partners.

This is just one example of many. Experiences gained over six years with COMDIS-HSD show that the path to publication for NGOs can be a murky and complex process riddled with maddening bureaucracy on one end of the spectrum, to a slightly more transparent and swifter process on the other. The NGO sector is not alone in experiencing frustration with APC payment mechanisms. Interviews with both librarians and publishers highlighted systems that were “extremely challenging and inefficient” ( Björk & Soloman, 2014).

## Summary

In consortia that rely on Northern universities partnering with Southern NGOs, NGO efforts to access and publish papers are being funnelled through their Northern counterparts. This creates an unnecessary dependency on the privileges afforded to Northern universities to access relevant research literature, and an over-reliance on Northern university partners to help negotiate and secure APC waivers and pay APCs.

Ultimately, NGOs operating in LMICs experience more friction than their Northern university partners in accessing research and in publishing their research findings. What this shows is that NGO partners in LMICs are less supported by publishers and funders to access research and to make their work accessible. This points to a fundamental lack of understanding about NGO organisational structures and their operational practices and protocols, as well as a lack of understanding of their value in the research evidence production cycle.

Furthermore, NGOs in the Global South remain excluded from open access debates and unrepresented in planning and advisory committees, particularly those convened by funders and Northern universities and research institutes, for example, Coalition S Ambassadors, Research4Life partners, and FORCE11 advisors. Their exclusion is an example of how knowledge access inequalities are embedded in the research production cycle. To redress this, a step forward would be to actively include these important research organisations from the Global South in OA discussions and implementation plans.

## Data availability

No data are associated with this article.
