# Supplementary material for: NGOs’ experiences of navigating the open access landscape
Source: F1000Res. 2019 Oct 18;8:1563. Originally published 2019 Sep 2. [Version 2] doi: 10.12688/f1000research.17359.2 (PMC6880624; doi:10.12688/f1000research.17359.2)
Supplement: Peer review history

# Peer review: Reviewer response for version 2

**DOI:** 10.5256/f1000research.23013.r55870

**Published:** 2019-11-21

**Authors:** Samir Hachani

**Affiliations:** 1 Algiers' University, Algiers, Algeria

Thanks for the author for a very insightful article. Open access in the Global South is very important issues as it may alleviate the digital divide that is preponderant in today’s publishing world .On the other hand, NGOs (especially those that are local) represent a viable and unavoidable partner for a fruitful cooperation between the Global North and the Global South. This article has adequately presented the issue at stake and has pinpointed the inadequacies the system holds. Being from the Global South, I can relate to the issues the author is investigating especially those pertaining to the APCs. In our countries, the sums asked simply represents well over one month of salary which make them not only expensive but simply impossible. However, I think that the author could have added some references that have investigated the issues and I think these two are really at the heart of the subject at hand. They are:

1. Dahdouh-Faridet *et al.*, (2003), Neo colonial science by the most industrialized upon the least developed countries in peer reviewed publication, *Scientometrics*, 56(3), 329-343 ^1^.
2. Benjamin Acosta-Cazares *et al. *(2000).Scientific Colonialism and Safari Research. Available here ^2^.

They show how much scientific publishing has not earned its independence from outdated and inefficient ways to publish and that have made publishing some kind of “old boys’ network” only that the network relates not to people individually but groups that are simply eliminated from publishing not intrinsically but on a personal base. That’s where the author could have made the case (she did but not strongly enough) that the exclusion they (we?) suffer is embedded in the system that do not take account large chunks of potential contributors to knowledge building. I commend the author last sentences “ *Their exclusion is an example of how knowledge access inequalities are embedded in the research production cycle. To redress this, a step forward would be to actively include these important research organisations from the Global South in OA discussions and implementation plans”*. It perfectly summarises an inequitable system that exclude on non logical and rather biased criteria.

 One last remark: the keywords include Nepal, Bangladesh, China, Pakistan, Swaziland, Ghana but only Nepal’s example is cited inside the text. Is this an omission or these countries were studied but not namely in the article?

I confirm that I have read this submission and believe that I have an appropriate level of expertise to confirm that it is of an acceptable scientific standard.

## References

[1] : *Scientometrics*.2003;56(3) : 10.1023/A:1022374703178 329-343 10.1023/A:1022374703178

[2] : Scientific Colonialism and Safari Research.2000; Reference source

# Peer review: Reviewer response for version 2

**DOI:** 10.5256/f1000research.23013.r55425

**Published:** 2019-10-28

**Authors:** Ross Mounce

**Affiliations:** 1 Arcadia Fund, London, UK

I am pleased with how the author has responded to my comments on the first version of this manuscript.

 The changes and the explanation of those changes in the author response satisfy me.

 One very minor thing, which I regret I did not spot or discuss on the first version of the manuscript:

 In April 2018, the official name of 'Swaziland' was changed from Kingdom of Swaziland to Kingdom of Eswatini sz. So the keywords for this manuscript should perhaps include 'Eswatini' rather than 'Swaziland'. Although I guess one could argue if the work was done pre-2018 when the country was called Swaziland perhaps calling it that better reflects the time-period?

I confirm that I have read this submission and believe that I have an appropriate level of expertise to confirm that it is of an acceptable scientific standard.

# Peer review: Reviewer response for version 1

**DOI:** 10.5256/f1000research.18984.r53316

**Published:** 2019-09-16

**Authors:** Ross Mounce

**Affiliations:** 1 Arcadia Fund, London, UK

I thank the author for this insightful opinion article documenting her experience of navigating the open access publishing landscape with NGOs. There are some really novel insights in here which to my knowledge have not yet been communicated in a peer-reviewed forum, so thanks to the author for taking the time to do so. The anecdotes contained within this article are extremely important to be conveyed and to be more widely known amongst the Global North research, publishing and policy communities who may appear otherwise sometimes oblivious to many of the problems discussed here.

 Here are some small constructive comments:

1. "However, publishers incorrectly viewed the UK base of a corresponding author as an indication of the ability to pay the full open access APC, adding a value added tax of 20% to the original cost."
  With respect, I think this conflates two slightly different issues. If an APC is being paid from the UK, then legally-speaking Value Added Tax (which is currently set at 20%) has to be paid, and it not the publisher that is mandating this, but instead the UK government. If the APC could be paid by another author not based in the UK, not from a UK bank account, then that author/institution/NGO might not need to pay UK VAT. The second problem intermingled is the publisher assuming that because a corresponding author affiliation appears to be from the UK, that they somehow automatically have the money to pay a full APC.
  Both are legitimate problems.
  But I just think they need to be teased apart slightly to make it clear that there are two problems there not just one. In defence of the publishers, sometimes APCs are known to be invoiced on the basis of who is indicated as the corresponding author of the paper. For some publishers it doesn't matter if the majority of the authors are from LMICs, all that matters is who is the corresponding author. Nilam is right to highlight this as a seemingly unjust problem. To be clear I am not defending publisher policies here (far from it) but if the policy is transparent as least NGOs and authors might know about it before submitting.
  For example BMC (owned by Springer Nature) write: "BMC offers APC waivers to papers whose corresponding authors are based in countries classified by the World Bank as low-income economies as of July 2019." https://www.biomedcentral.com/getpublished/article-processing-charges/open-access-waiver-fund 
2. Perhaps the author might consider referring to 'research outputs' rather 'research evidence'? From my point of view in the funder landscape I feel that outputs is the more common term but perhaps there is a specific reason for using 'evidence' rather than outputs? Something like the production of scientific software might not be considered 'research evidence' but would clearly be a 'research output'.
3. In the discussion of those in the Global South needing to use Sci-Hub, I would perhaps cite voices from the Global South to support this point such as Bendezú-Quispe *et al.* 2016 "Sci-Hub and medical practice: an ethical dilemma in Peru" ^1^.
  The well-documented above average intensity of usage of Sci-Hub in Iran might also be worth citing on this point with respect to the Global South (Greshake, 2016 "Correlating the Sci-Hub data with World Bank Indicators and Identifying Academic Use" ^2^).
4. In general, whilst it is important that Nilam's personal experience makes up the majority of this opinion article, it would be good to include many further references on what others have said about similar problems with the open access landscape such as:
  Peterson, A.; Emmett, A.; Greenberg, M.L. Open access and the author-pays problem: Assuring access for readers and authors in a global community of scholars. J. Librariansh. Sch. Commun. 2013 ^3^
  Lawson (2015) "Fee Waivers for Open Access Journals" https://doi.org/10.3390/publications3030155 ^4^
  Bonaccorso, E., Bozhankova, R., Cadena, C., Čapská, V., Czerniewicz, L., Emmett, A., Oludayo, F., Glukhova, N., *et al.* 2014. Bottlenecks in the Open-Access System: Voices from Around the Globe. ^5^
  Jørgen Burchardt (2014) Researchers Outside APC-Financed Open Access: Implications for Scholars Without a Paying Institution https://doi.org/10.1177/2158244014551714 ^6^
5. Finally, as Nilam has been working with Health NGOs, I do think a citation to statements in this paper is warranted...
  "In fact, hybrid OA journals, which have the most expensive APCs, were the most underrepresented in LMICs. It remains unclear why APCs for hybrid journals remain higher than gold APCs given the fact that these journals also ask for subscription fees.
  Since the APCs are mainly paid to the ten same publishers creating an oligopoly, there is little incentive to keep APCs low. This oligopoly may also run much deeper than costs; it creates an important inequity in publication. Although publishers may wish to include researchers from LMICs through waivers, they have not really included LMICs in the publication industry itself. "
  -- Smith *et al *(2017) "Knowledge sharing in global health research – the impact, uptake and cost of open access to scholarly literature" Health Research Policy and Systems ^7^

I confirm that I have read this submission and believe that I have an appropriate level of expertise to confirm that it is of an acceptable scientific standard, however I have significant reservations, as outlined above.

## References

[1] : Sci-Hub and medical practice: an ethical dilemma in Peru. *Lancet Glob Health*.4(9) : 10.1016/S2214-109X(16)30188-7 e608 10.1016/S2214-109X(16)30188-7 27539805

[2] : Correlating the Sci-Hub data with World Bank Indicators and Identifying Academic Use. *The Winnower*.2016; 10.15200/winn.146485.57797 10.15200/winn.146485.57797

[3] : Open Access and the Author-Pays Problem: Assuring Access for Readers and Authors in the Global Academic Community. *Journal of Librarianship and Scholarly Communication*.2013;1(3) : 10.7710/2162-3309.1064 10.7710/2162-3309.1064

[4] : Fee Waivers for Open Access Journals. *Publications*.2015;3(3) : 10.3390/publications3030155 155-167 10.3390/publications3030155

[5] : Bottlenecks in the Open-Access System: Voices from Around the Globe. *Journal of Librarianship and Scholarly Communication*.2014;2(2) : 10.7710/2162-3309.1126 10.7710/2162-3309.1126

[6] : Researchers Outside APC-Financed Open Access. *SAGE Open*.2014;4(4) : 10.1177/2158244014551714 10.1177/2158244014551714

[7] : Knowledge sharing in global health research - the impact, uptake and cost of open access to scholarly literature. *Health Res Policy Syst*.2017;15(1) : 10.1186/s12961-017-0235-3 73 10.1186/s12961-017-0235-3 28851401PMC5576373

# Response

**Published:** 2019-10-14

**Authors:** Nilam McGrath

**Affiliations:** Independent, UK

Dear Ross,

Many thanks for taking the time to provide an extended review - I appreciate it. Below is my response to the points you raise:

- I agree that I have conflated two ideas and I have now rewritten the section so that the two separate points are clearer. The rewrite has allowed me to elaborate on the waiver inconsistencies as experienced by COMDIS-HSD and to support the experiences by citing recent studies (thank you for signposting to these papers).
- The distinction between ‘research evidence’ and ‘research outputs’ is a useful one. There are clearly instances in this article where changing the text to ‘research outputs’ or ‘journal articles’ lends clarity to the argument and I have changed the text where relevant. In a small number of instances, I have retained the use of ‘research evidence’ where this refers to the research production cycle.
- I have expanded the section on Sci-Hub, citing references that highlight its popularity in China, India and Iran, and the ethical dilemma that researchers for researchers.
- I have cited recent studies that support the COMDIS-HSD experience, specifically: the inequality of knowledge production and sharing; a more detailed explanation of Research4Life’s offer and how this relates to the nature of competing funding priorities for health NGOs (including a pertinent quote from Smith *et al *2017); further references to the popularity of Sci-Hub; and differing waiver and payment processes across publishers. Again, thank you for signposting to these studies.
- I have also made minor edits to the text, e.g consistent use of ‘OA’ throughout.

Thanks again for your thoughtful, detailed and constructive review. I hope that the changes I have made fully address your reviewer comments.
